# *N*-[2-(5-Bromo-2-morpholin-4-ylpyrim­idin-4-ylsulfan­yl)-4-meth­oxy­phen­yl]-2,4,6-trimethyl­benzene­sulfonamide

**DOI:** 10.1107/S1600536812040792

**Published:** 2012-10-03

**Authors:** Mohan Kumar, L. Mallesha, M. A. Sridhar, Kamini Kapoor, Vivek K. Gupta, Rajni Kant

**Affiliations:** a Department of Studies in Physics, Manasagangotri, University of Mysore, Mysore 570 006, India; b PG Department of Studies in Chemistry, JSS College of Arts, Commerce and Science, Ooty Road, Mysore 570 025, India; c X-ray Crystallography Laboratory, Post-Graduate Department of Physics & Electronics, University of Jammu, Jammu Tawi 180 006, India

## Abstract

In the title compound, C_24_H_27_BrN_4_O_4_S_2_, the mol­ecule is twisted at the sulfonyl S atom with a C—S(O_2_)—N(H)—C torsion angle of 62.6 (3)°. The benzene rings bridged by the sulfonamide group are tilted to each other by a dihedral angle of 60.6 (1)°. The dihedral angle between the sulfur-bridged pyrimidine and benzene rings is 62.7 (1)°. The morpholine ring adopts a chair conformation. The mol­ecular conformation is stabilized by a weak intra­molecular π–π stacking inter­action between the pyrimidine and the 2,4,6-trimethyl­benzene rings [centroid–centroid distance = 3.793 (2) Å]. In the crystal, mol­ecules are linked by N—H⋯O hydrogen bonds into a chain along the *b* axis.

## Related literature

For related structures of sulfonamides, see: Rodrigues *et al.* (2011 ▶); Akkurt *et al.* (2011 ▶); Kant *et al.* (2012 ▶). For bond-length data, see: Allen *et al.* (1987 ▶). For ring conformations, see: Duax & Norton (1975 ▶).
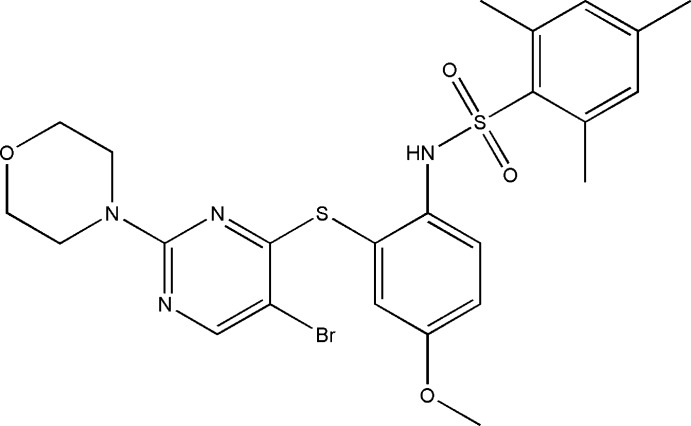


## Experimental

### 

#### Crystal data

C_24_H_27_BrN_4_O_4_S_2_
*M* *_r_* = 579.53
Monoclinic, 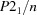

*a* = 10.2583 (4) Å
*b* = 17.4727 (6) Å
*c* = 14.4375 (7) Å
β = 97.199 (4)°
*V* = 2567.38 (18) Å^3^
*Z* = 4
Mo *K*α radiation
μ = 1.80 mm^−1^
*T* = 293 K
0.3 × 0.2 × 0.2 mm

#### Data collection

Oxford Diffraction Xcalibur Sapphire3 diffractometer
Absorption correction: multi-scan (*CrysAlis PRO*; Oxford Diffraction, 2010 ▶) *T*_min_ = 0.526, *T*_max_ = 0.697
19056 measured reflections
5023 independent reflections
3359 reflections with *I* > 2σ(*I*)
*R*_int_ = 0.045

#### Refinement

*R*[*F* ^2^ > 2σ(*F* ^2^)] = 0.046
*wR*(*F* ^2^) = 0.105
*S* = 1.02
5023 reflections
321 parameters
H-atom parameters constrained
Δρ_max_ = 0.31 e Å^−3^
Δρ_min_ = −0.39 e Å^−3^

### 

Data collection: *CrysAlis PRO* (Oxford Diffraction, 2010 ▶); cell refinement: *CrysAlis PRO*; data reduction: *CrysAlis PRO*; program(s) used to solve structure: *SHELXS97* (Sheldrick, 2008 ▶); program(s) used to refine structure: *SHELXL97* (Sheldrick, 2008 ▶); molecular graphics: *ORTEP-3* (Farrugia, 1997 ▶); software used to prepare material for publication: *PLATON* (Spek, 2009 ▶).

## Supplementary Material

Crystal structure: contains datablock(s) I, global. DOI: 10.1107/S1600536812040792/is5200sup1.cif

Structure factors: contains datablock(s) I. DOI: 10.1107/S1600536812040792/is5200Isup2.hkl

Supplementary material file. DOI: 10.1107/S1600536812040792/is5200Isup3.cml

Additional supplementary materials:  crystallographic information; 3D view; checkCIF report

## Figures and Tables

**Table 1:** Hydrogen-bond geometry (Å, °)

*D*—H⋯*A*	*D*—H	H⋯*A*	*D*⋯*A*	*D*—H⋯*A*
N1—H1⋯O25^i^	0.86	2.09	2.890 (4)	155

Symmetry code: (i) 
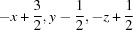
.

**Fig. 1.**
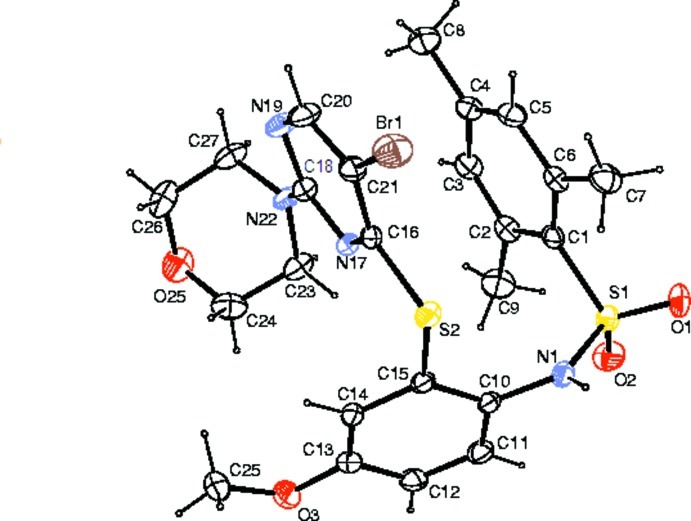
ORTEP view of the molecule with the atom-labeling scheme. The displacement ellipsoids are drawn at the 40% probability level. H atoms are shown as small spheres of arbitrary radii.

**Fig. 2.**
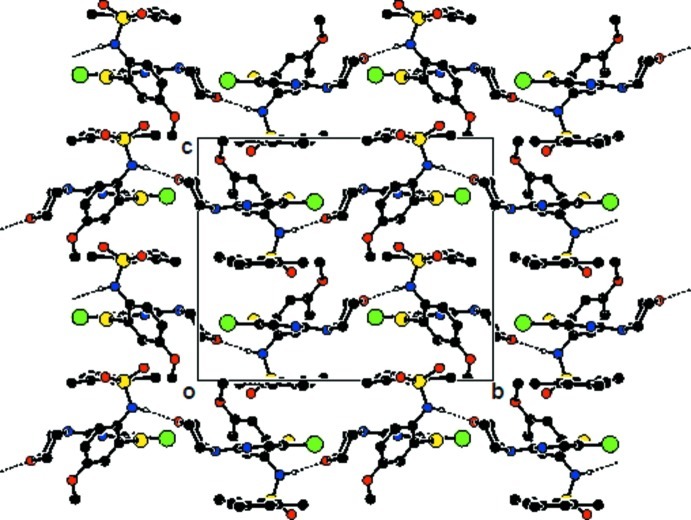
A molecular packing view of the title compound down the a axis, showing intermolecular interactions (dashed lines). For clarity, H atoms not involved in the hydrogen bonds have been omitted.

## supplementary crystallographic information

### Comment

Bond lengths and angles in the title compound (Fig. 1) have normal values (Allen *et al.*, 1987) and are comparable with the similar crystal structures (Rodrigues *et al.*, 2011; Akkurt *et al.*, 2011; Kant *et al.*, 2012). The molecule is twisted at the S atom with the C2—S1—N1—C10 torsion angle of 62.6 (3)°. The morpholine ring is exhibiting chair conformation (asymmetry parameters are: [ΔC2(N22—C27) = 2.51; ΔCs(C24—C27) = 0.92] (Duax & Norton, 1975). The benzene C1–C6 and C10–C15 rings are tilted relative to each other by 60.6 (1)° and the dihedral angle between the sulfur bridged pyrimidine and benzene rings is 62.7 (1) °. The molecular conformation is stabilized by a weak intramolecular π–π stacking interaction between the pyrimidine C16/N17/C18/N19/C20/C21 ring (centroid *Cg*1) and the 2,4,6-trimethyl benzene C1–C6 ring (centroid *Cg*2) [*Cg*1···*Cg*2 3.793 (2) Å, perpendicular distance of *Cg*1 on the C1–C6 ring 3.5103 (13) Å and perpendicular distance of *Cg*2 on the C16/N17/C18/N19/C20/C21 ring 3.3905 (12) Å]. In the crystal, molecules are linked into chains by N1—H1···O25 hydrogen bonds (Table 1 and Fig. 2).

### Experimental

The reaction of *N*-[2-(5-bromo-2-morpholin-4-yl-pyrimidin-4-ylsulfanyl)-4-methoxy-phenyl]-2,4,6-trimethyl-benzenesulfonamide (5.29 g, 0.01 mol) with morpholine (0.88 g, 0.01 mol) were carried out in the presence of triethylamine and the reaction mixture was allowed to stir at room temperature for 6–7 h in dry dichloromethane. The progress of the reaction was monitored by TLC. Upon completion, the solvent was removed under reduced pressure and residue was extracted with ethyl acetate. The compound was purified by successive recrystallization from methanol (yield 83%, m.p. 454–456 K).

### Refinement

All H atoms were positioned geometrically and were treated as riding on their parent C/N atoms, with C—H = 0.93–0.97 Å and N—H = 0.86 Å, and with *U*_iso_(H) = 1.2*U*_eq_(C) or 1.5*U*_eq_(methyl C).

### Crystal data

**Table:**

C_24_H_27_BrN_4_O_4_S_2_	*F*(000) = 1192
*M**_r_* = 579.53	*D*_x_ = 1.499 Mg m^−^^3^
Monoclinic, *P*2_1_/*n*	Mo *K*α radiation, λ = 0.71073 Å
Hall symbol: -P 2yn	Cell parameters from 6670 reflections
*a* = 10.2583 (4) Å	θ = 3.7–29.0°
*b* = 17.4727 (6) Å	µ = 1.80 mm^−^^1^
*c* = 14.4375 (7) Å	*T* = 293 K
β = 97.199 (4)°	Block, brown
*V* = 2567.38 (18) Å^3^	0.3 × 0.2 × 0.2 mm
*Z* = 4	

### Data collection

**Table:**

Oxford Diffraction Xcalibur Sapphire3 diffractometer	5023 independent reflections
Radiation source: fine-focus sealed tube	3359 reflections with *I* > 2σ(*I*)
Graphite monochromator	*R*_int_ = 0.045
Detector resolution: 16.1049 pixels mm^-1^	θ_max_ = 26.0°, θ_min_ = 3.7°
ω scan	*h* = −12→12
Absorption correction: multi-scan (*CrysAlis PRO*; Oxford Diffraction, 2010)	*k* = −21→21
*T*_min_ = 0.526, *T*_max_ = 0.697	*l* = −17→17
19056 measured reflections	

### Refinement

**Table:**

Refinement on *F*^2^	Secondary atom site location: difference Fourier map
Least-squares matrix: full	Hydrogen site location: inferred from neighbouring sites
*R*[*F*^2^ > 2σ(*F*^2^)] = 0.046	H-atom parameters constrained
*wR*(*F*^2^) = 0.105	*w* = 1/[σ^2^(*F*_o_^2^) + (0.0338*P*)^2^ + 1.6315*P*] where *P* = (*F*_o_^2^ + 2*F*_c_^2^)/3
*S* = 1.02	(Δ/σ)_max_ = 0.001
5023 reflections	Δρ_max_ = 0.31 e Å^−^^3^
321 parameters	Δρ_min_ = −0.39 e Å^−^^3^
0 restraints	Extinction correction: *SHELXL*, Fc^*^=kFc[1+0.001xFc^2^λ^3^/sin(2θ)]^-1/4^
Primary atom site location: structure-invariant direct methods	Extinction coefficient: 0.0057 (4)

### Special details

**Table:**

Experimental. *CrysAlis PRO*, Oxford Diffraction Ltd., Version 1.171.34.40 (release 27–08-2010 CrysAlis171. NET) (compiled Aug 27 2010,11:50:40) Empirical absorption correction using spherical harmonics, implemented in SCALE3 ABSPACK scaling algorithm.
Geometry. All e.s.d.'s (except the e.s.d. in the dihedral angle between two l.s. planes) are estimated using the full covariance matrix. The cell e.s.d.'s are taken into account individually in the estimation of e.s.d.'s in distances, angles and torsion angles; correlations between e.s.d.'s in cell parameters are only used when they are defined by crystal symmetry. An approximate (isotropic) treatment of cell e.s.d.'s is used for estimating e.s.d.'s involving l.s. planes.
Refinement. Refinement of *F*^2^ against ALL reflections. The weighted *R*-factor *wR* and goodness of fit *S* are based on *F*^2^, conventional *R*-factors *R* are based on *F*, with *F* set to zero for negative *F*^2^. The threshold expression of *F*^2^ > σ(*F*^2^) is used only for calculating *R*-factors(gt) *etc*. and is not relevant to the choice of reflections for refinement. *R*-factors based on *F*^2^ are statistically about twice as large as those based on *F*, and *R*- factors based on ALL data will be even larger.

### Fractional atomic coordinates and isotropic or equivalent isotropic displacement parameters (Å^2^)

**Table:**

	*x*	*y*	*z*	*U*_iso_*/*U*_eq_	
Br1	1.02664 (4)	0.10295 (2)	0.23807 (3)	0.07706 (19)	
S1	0.50455 (9)	0.24940 (6)	0.00299 (6)	0.0566 (3)	
S2	0.74901 (8)	0.19238 (5)	0.24649 (6)	0.0472 (2)	
N1	0.5008 (2)	0.22120 (16)	0.11087 (18)	0.0487 (7)	
H1	0.4848	0.1739	0.1215	0.058*	
O1	0.4808 (3)	0.18139 (17)	−0.05220 (17)	0.0788 (8)	
O2	0.4152 (2)	0.31164 (17)	−0.01409 (18)	0.0774 (8)	
O3	0.5639 (3)	0.42389 (15)	0.41145 (19)	0.0704 (7)	
C1	0.6672 (3)	0.28269 (18)	−0.0041 (2)	0.0430 (8)	
C2	0.6972 (4)	0.36148 (19)	−0.0014 (2)	0.0508 (9)	
C3	0.8256 (4)	0.3831 (2)	−0.0079 (2)	0.0562 (9)	
H3	0.8455	0.4350	−0.0071	0.067*	
C4	0.9252 (3)	0.3316 (2)	−0.0154 (2)	0.0535 (9)	
C5	0.8941 (3)	0.2553 (2)	−0.0154 (2)	0.0527 (9)	
H5	0.9608	0.2197	−0.0187	0.063*	
C6	0.7666 (3)	0.22861 (18)	−0.0106 (2)	0.0460 (8)	
C7	0.7498 (4)	0.14289 (19)	−0.0125 (3)	0.0681 (11)	
H7A	0.7078	0.1274	−0.0727	0.102*	
H7B	0.6966	0.1278	0.0346	0.102*	
H7C	0.8344	0.1188	−0.0005	0.102*	
C8	1.0642 (4)	0.3581 (3)	−0.0207 (3)	0.0791 (12)	
H8A	1.0826	0.3555	−0.0842	0.119*	
H8B	1.1244	0.3257	0.0176	0.119*	
H8C	1.0740	0.4100	0.0011	0.119*	
C9	0.6004 (4)	0.4257 (2)	0.0093 (3)	0.0787 (13)	
H9A	0.5321	0.4249	−0.0427	0.118*	
H9B	0.6453	0.4739	0.0110	0.118*	
H9C	0.5625	0.4187	0.0662	0.118*	
C10	0.5231 (3)	0.27276 (18)	0.1873 (2)	0.0412 (7)	
C11	0.4332 (3)	0.3308 (2)	0.1984 (2)	0.0532 (9)	
H11	0.3594	0.3360	0.1544	0.064*	
C12	0.4508 (3)	0.3801 (2)	0.2724 (3)	0.0553 (9)	
H12	0.3902	0.4191	0.2774	0.066*	
C13	0.5583 (3)	0.3723 (2)	0.3399 (2)	0.0503 (8)	
C14	0.6476 (3)	0.31466 (18)	0.3324 (2)	0.0446 (8)	
H14	0.7183	0.3082	0.3787	0.054*	
C15	0.6321 (3)	0.26588 (18)	0.2552 (2)	0.0409 (7)	
C16	0.8899 (3)	0.24701 (19)	0.2337 (2)	0.0404 (7)	
N17	0.8793 (2)	0.32166 (15)	0.22635 (17)	0.0414 (6)	
C18	0.9896 (3)	0.3618 (2)	0.2209 (2)	0.0490 (8)	
N19	1.1113 (3)	0.33241 (18)	0.2232 (2)	0.0611 (8)	
C20	1.1185 (3)	0.2570 (2)	0.2294 (3)	0.0614 (10)	
H20	1.2006	0.2341	0.2313	0.074*	
C21	1.0110 (3)	0.2104 (2)	0.2331 (2)	0.0504 (8)	
N22	0.9747 (3)	0.43869 (16)	0.2097 (2)	0.0575 (8)	
C23	0.8513 (3)	0.4755 (2)	0.2241 (3)	0.0623 (10)	
H23A	0.7806	0.4382	0.2173	0.075*	
H23B	0.8303	0.5154	0.1780	0.075*	
C24	0.8650 (4)	0.5089 (2)	0.3196 (3)	0.0726 (12)	
H24A	0.7834	0.5335	0.3300	0.087*	
H24B	0.8829	0.4685	0.3655	0.087*	
O25	0.9689 (3)	0.56335 (15)	0.3307 (2)	0.0848 (9)	
C25	0.6630 (4)	0.4143 (2)	0.4887 (3)	0.0773 (12)	
H25A	0.6544	0.3647	0.5158	0.116*	
H25B	0.6535	0.4531	0.5345	0.116*	
H25C	0.7479	0.4187	0.4679	0.116*	
C26	1.0905 (4)	0.5289 (2)	0.3180 (3)	0.0808 (14)	
H26A	1.1128	0.4909	0.3663	0.097*	
H26B	1.1589	0.5676	0.3241	0.097*	
C27	1.0850 (3)	0.4917 (2)	0.2245 (3)	0.0679 (11)	
H27A	1.0756	0.5306	0.1761	0.082*	
H27B	1.1663	0.4643	0.2204	0.082*	

### Atomic displacement parameters (Å^2^)

**Table:**

	*U*^11^	*U*^22^	*U*^33^	*U*^12^	*U*^13^	*U*^23^
Br1	0.0695 (3)	0.0623 (3)	0.1013 (4)	0.0199 (2)	0.0181 (2)	0.0000 (2)
S1	0.0426 (5)	0.0840 (7)	0.0398 (5)	−0.0032 (5)	−0.0080 (4)	−0.0043 (5)
S2	0.0379 (4)	0.0491 (5)	0.0532 (5)	−0.0001 (4)	0.0004 (4)	−0.0006 (4)
N1	0.0435 (16)	0.0588 (17)	0.0423 (16)	−0.0127 (14)	−0.0004 (12)	−0.0017 (14)
O1	0.0723 (18)	0.108 (2)	0.0522 (16)	−0.0252 (16)	−0.0088 (13)	−0.0267 (16)
O2	0.0485 (15)	0.118 (2)	0.0611 (17)	0.0205 (15)	−0.0109 (12)	0.0180 (16)
O3	0.0646 (17)	0.0829 (18)	0.0653 (18)	0.0025 (14)	0.0142 (14)	−0.0251 (15)
C1	0.0422 (18)	0.056 (2)	0.0294 (16)	0.0062 (16)	0.0000 (14)	−0.0014 (15)
C2	0.062 (2)	0.048 (2)	0.043 (2)	0.0115 (17)	0.0059 (17)	0.0018 (16)
C3	0.072 (3)	0.046 (2)	0.053 (2)	−0.0036 (19)	0.0172 (19)	0.0023 (17)
C4	0.056 (2)	0.062 (2)	0.045 (2)	−0.0007 (19)	0.0162 (16)	0.0007 (18)
C5	0.053 (2)	0.054 (2)	0.052 (2)	0.0127 (17)	0.0136 (17)	−0.0006 (17)
C6	0.057 (2)	0.0443 (18)	0.0370 (18)	0.0048 (16)	0.0079 (15)	−0.0056 (15)
C7	0.080 (3)	0.050 (2)	0.078 (3)	0.002 (2)	0.024 (2)	−0.011 (2)
C8	0.068 (3)	0.090 (3)	0.083 (3)	−0.013 (2)	0.024 (2)	−0.002 (2)
C9	0.079 (3)	0.061 (2)	0.097 (3)	0.027 (2)	0.014 (2)	0.000 (2)
C10	0.0296 (16)	0.0559 (19)	0.0381 (17)	−0.0055 (15)	0.0044 (13)	0.0027 (16)
C11	0.0321 (18)	0.072 (2)	0.054 (2)	0.0021 (17)	0.0027 (15)	0.006 (2)
C12	0.040 (2)	0.067 (2)	0.061 (2)	0.0101 (17)	0.0152 (17)	−0.003 (2)
C13	0.043 (2)	0.061 (2)	0.049 (2)	−0.0048 (17)	0.0149 (16)	−0.0064 (18)
C14	0.0362 (17)	0.062 (2)	0.0356 (18)	−0.0064 (16)	0.0027 (14)	0.0004 (16)
C15	0.0289 (16)	0.0535 (19)	0.0403 (18)	−0.0021 (14)	0.0043 (13)	0.0047 (15)
C16	0.0340 (17)	0.056 (2)	0.0294 (16)	0.0019 (15)	−0.0008 (12)	−0.0041 (15)
N17	0.0294 (14)	0.0520 (17)	0.0424 (15)	0.0012 (12)	0.0025 (11)	−0.0056 (13)
C18	0.0378 (19)	0.058 (2)	0.050 (2)	0.0022 (17)	0.0043 (15)	−0.0052 (17)
N19	0.0327 (16)	0.068 (2)	0.084 (2)	0.0034 (15)	0.0117 (15)	−0.0023 (18)
C20	0.0334 (19)	0.074 (3)	0.079 (3)	0.0102 (19)	0.0130 (18)	−0.001 (2)
C21	0.0423 (19)	0.058 (2)	0.051 (2)	0.0100 (17)	0.0057 (15)	−0.0047 (17)
N22	0.0324 (15)	0.0558 (18)	0.085 (2)	−0.0048 (14)	0.0119 (14)	−0.0099 (16)
C23	0.041 (2)	0.049 (2)	0.096 (3)	0.0003 (17)	0.007 (2)	0.002 (2)
C24	0.078 (3)	0.054 (2)	0.092 (3)	0.013 (2)	0.033 (2)	0.010 (2)
O25	0.081 (2)	0.0571 (17)	0.113 (3)	0.0073 (16)	−0.0002 (18)	−0.0187 (16)
C25	0.079 (3)	0.100 (3)	0.055 (2)	−0.021 (2)	0.014 (2)	−0.026 (2)
C26	0.065 (3)	0.057 (2)	0.112 (4)	−0.005 (2)	−0.022 (3)	0.001 (3)
C27	0.040 (2)	0.061 (2)	0.104 (3)	−0.0081 (18)	0.014 (2)	0.008 (2)

### Geometric parameters (Å, º)

**Table:**

Br1—C21	1.885 (3)	C11—C12	1.368 (5)
S1—O2	1.424 (3)	C11—H11	0.9300
S1—O1	1.434 (3)	C12—C13	1.384 (5)
S1—N1	1.639 (3)	C12—H12	0.9300
S1—C1	1.782 (3)	C13—C14	1.376 (4)
S2—C16	1.761 (3)	C14—C15	1.396 (4)
S2—C15	1.772 (3)	C14—H14	0.9300
N1—C10	1.421 (4)	C16—N17	1.312 (4)
N1—H1	0.8600	C16—C21	1.399 (4)
O3—C13	1.367 (4)	N17—C18	1.342 (4)
O3—C25	1.421 (5)	C18—N19	1.347 (4)
C1—C6	1.402 (4)	C18—N22	1.359 (4)
C1—C2	1.410 (4)	N19—C20	1.322 (4)
C2—C3	1.385 (5)	C20—C21	1.377 (5)
C2—C9	1.519 (5)	C20—H20	0.9300
C3—C4	1.375 (5)	N22—C27	1.457 (4)
C3—H3	0.9300	N22—C23	1.458 (4)
C4—C5	1.371 (5)	C23—C24	1.487 (5)
C4—C8	1.511 (5)	C23—H23A	0.9700
C5—C6	1.398 (5)	C23—H23B	0.9700
C5—H5	0.9300	C24—O25	1.423 (5)
C6—C7	1.508 (4)	C24—H24A	0.9700
C7—H7A	0.9600	C24—H24B	0.9700
C7—H7B	0.9600	O25—C26	1.417 (5)
C7—H7C	0.9600	C25—H25A	0.9600
C8—H8A	0.9600	C25—H25B	0.9600
C8—H8B	0.9600	C25—H25C	0.9600
C8—H8C	0.9600	C26—C27	1.493 (6)
C9—H9A	0.9600	C26—H26A	0.9700
C9—H9B	0.9600	C26—H26B	0.9700
C9—H9C	0.9600	C27—H27A	0.9700
C10—C11	1.393 (4)	C27—H27B	0.9700
C10—C15	1.396 (4)		
			
O2—S1—O1	118.40 (17)	O3—C13—C12	115.0 (3)
O2—S1—N1	107.62 (16)	C14—C13—C12	119.8 (3)
O1—S1—N1	104.85 (16)	C13—C14—C15	120.0 (3)
O2—S1—C1	109.14 (16)	C13—C14—H14	120.0
O1—S1—C1	109.49 (16)	C15—C14—H14	120.0
N1—S1—C1	106.67 (13)	C14—C15—C10	120.6 (3)
C16—S2—C15	100.73 (15)	C14—C15—S2	119.2 (2)
C10—N1—S1	121.8 (2)	C10—C15—S2	120.1 (2)
C10—N1—H1	119.1	N17—C16—C21	121.2 (3)
S1—N1—H1	119.1	N17—C16—S2	119.1 (2)
C13—O3—C25	118.3 (3)	C21—C16—S2	119.6 (3)
C6—C1—C2	120.1 (3)	C16—N17—C18	117.6 (3)
C6—C1—S1	118.6 (2)	N17—C18—N19	125.8 (3)
C2—C1—S1	121.3 (2)	N17—C18—N22	116.2 (3)
C3—C2—C1	118.0 (3)	N19—C18—N22	118.1 (3)
C3—C2—C9	116.5 (3)	C20—N19—C18	115.2 (3)
C1—C2—C9	125.5 (3)	N19—C20—C21	123.6 (3)
C4—C3—C2	123.3 (3)	N19—C20—H20	118.2
C4—C3—H3	118.3	C21—C20—H20	118.2
C2—C3—H3	118.3	C20—C21—C16	116.5 (3)
C5—C4—C3	117.4 (3)	C20—C21—Br1	121.7 (3)
C5—C4—C8	121.3 (3)	C16—C21—Br1	121.8 (3)
C3—C4—C8	121.3 (3)	C18—N22—C27	122.5 (3)
C4—C5—C6	122.9 (3)	C18—N22—C23	120.3 (3)
C4—C5—H5	118.6	C27—N22—C23	111.8 (3)
C6—C5—H5	118.6	N22—C23—C24	108.8 (3)
C5—C6—C1	118.1 (3)	N22—C23—H23A	109.9
C5—C6—C7	115.8 (3)	C24—C23—H23A	109.9
C1—C6—C7	126.0 (3)	N22—C23—H23B	109.9
C6—C7—H7A	109.5	C24—C23—H23B	109.9
C6—C7—H7B	109.5	H23A—C23—H23B	108.3
H7A—C7—H7B	109.5	O25—C24—C23	110.5 (3)
C6—C7—H7C	109.5	O25—C24—H24A	109.6
H7A—C7—H7C	109.5	C23—C24—H24A	109.6
H7B—C7—H7C	109.5	O25—C24—H24B	109.6
C4—C8—H8A	109.5	C23—C24—H24B	109.6
C4—C8—H8B	109.5	H24A—C24—H24B	108.1
H8A—C8—H8B	109.5	C26—O25—C24	111.1 (3)
C4—C8—H8C	109.5	O3—C25—H25A	109.5
H8A—C8—H8C	109.5	O3—C25—H25B	109.5
H8B—C8—H8C	109.5	H25A—C25—H25B	109.5
C2—C9—H9A	109.5	O3—C25—H25C	109.5
C2—C9—H9B	109.5	H25A—C25—H25C	109.5
H9A—C9—H9B	109.5	H25B—C25—H25C	109.5
C2—C9—H9C	109.5	O25—C26—C27	111.6 (3)
H9A—C9—H9C	109.5	O25—C26—H26A	109.3
H9B—C9—H9C	109.5	C27—C26—H26A	109.3
C11—C10—C15	117.7 (3)	O25—C26—H26B	109.3
C11—C10—N1	120.4 (3)	C27—C26—H26B	109.3
C15—C10—N1	121.9 (3)	H26A—C26—H26B	108.0
C12—C11—C10	121.6 (3)	N22—C27—C26	110.5 (3)
C12—C11—H11	119.2	N22—C27—H27A	109.6
C10—C11—H11	119.2	C26—C27—H27A	109.6
C11—C12—C13	120.2 (3)	N22—C27—H27B	109.6
C11—C12—H12	119.9	C26—C27—H27B	109.6
C13—C12—H12	119.9	H27A—C27—H27B	108.1
O3—C13—C14	125.2 (3)		
			
O2—S1—N1—C10	−54.4 (3)	C13—C14—C15—C10	−3.0 (5)
O1—S1—N1—C10	178.7 (2)	C13—C14—C15—S2	179.5 (2)
C1—S1—N1—C10	62.6 (3)	C11—C10—C15—C14	1.5 (4)
O2—S1—C1—C6	−165.8 (2)	N1—C10—C15—C14	−176.3 (3)
O1—S1—C1—C6	−34.8 (3)	C11—C10—C15—S2	179.0 (2)
N1—S1—C1—C6	78.2 (3)	N1—C10—C15—S2	1.2 (4)
O2—S1—C1—C2	15.5 (3)	C16—S2—C15—C14	−65.8 (3)
O1—S1—C1—C2	146.6 (3)	C16—S2—C15—C10	116.7 (3)
N1—S1—C1—C2	−100.5 (3)	C15—S2—C16—N17	−4.9 (3)
C6—C1—C2—C3	1.8 (5)	C15—S2—C16—C21	174.0 (3)
S1—C1—C2—C3	−179.6 (2)	C21—C16—N17—C18	−1.8 (4)
C6—C1—C2—C9	−177.5 (3)	S2—C16—N17—C18	177.1 (2)
S1—C1—C2—C9	1.2 (5)	C16—N17—C18—N19	−0.7 (5)
C1—C2—C3—C4	−1.0 (5)	C16—N17—C18—N22	177.5 (3)
C9—C2—C3—C4	178.3 (3)	N17—C18—N19—C20	1.6 (5)
C2—C3—C4—C5	−0.7 (5)	N22—C18—N19—C20	−176.6 (3)
C2—C3—C4—C8	−179.0 (3)	C18—N19—C20—C21	0.1 (6)
C3—C4—C5—C6	1.8 (5)	N19—C20—C21—C16	−2.4 (5)
C8—C4—C5—C6	180.0 (3)	N19—C20—C21—Br1	178.1 (3)
C4—C5—C6—C1	−1.0 (5)	N17—C16—C21—C20	3.3 (5)
C4—C5—C6—C7	179.3 (3)	S2—C16—C21—C20	−175.6 (3)
C2—C1—C6—C5	−0.8 (5)	N17—C16—C21—Br1	−177.2 (2)
S1—C1—C6—C5	−179.5 (2)	S2—C16—C21—Br1	3.9 (4)
C2—C1—C6—C7	178.9 (3)	N17—C18—N22—C27	165.2 (3)
S1—C1—C6—C7	0.2 (4)	N19—C18—N22—C27	−16.4 (5)
S1—N1—C10—C11	67.5 (4)	N17—C18—N22—C23	13.2 (5)
S1—N1—C10—C15	−114.8 (3)	N19—C18—N22—C23	−168.4 (3)
C15—C10—C11—C12	0.7 (5)	C18—N22—C23—C24	98.4 (4)
N1—C10—C11—C12	178.5 (3)	C27—N22—C23—C24	−56.4 (4)
C10—C11—C12—C13	−1.4 (5)	N22—C23—C24—O25	59.2 (4)
C25—O3—C13—C14	−5.5 (5)	C23—C24—O25—C26	−60.5 (4)
C25—O3—C13—C12	173.0 (3)	C24—O25—C26—C27	57.3 (5)
C11—C12—C13—O3	−178.6 (3)	C18—N22—C27—C26	−100.5 (4)
C11—C12—C13—C14	−0.1 (5)	C23—N22—C27—C26	53.6 (4)
O3—C13—C14—C15	−179.3 (3)	O25—C26—C27—N22	−53.4 (4)
C12—C13—C14—C15	2.3 (5)		

### Hydrogen-bond geometry (Å, º)

**Table:**

*D*—H···*A*	*D*—H	H···*A*	*D*···*A*	*D*—H···*A*
N1—H1···O25^i^	0.86	2.09	2.890 (4)	155

Symmetry code: (i) −*x*+3/2, *y*−1/2, −*z*+1/2.

## References

[bb1] Akkurt, M., Mariam, I., Naseer, I., Khan, I. U. & Sharif, S. (2011). *Acta Cryst.* E**67**, o186. 10.1107/S1600536810052633 PMC3050146 21522690

[bb2] Allen, F. H., Kennard, O., Watson, D. G., Brammer, L., Orpen, A. G. & Taylor, R. (1987). *J. Chem. Soc. Perkin Trans. 2*, pp. S1–19.

[bb3] Duax, W. L. & Norton, D. A. (1975). *Atlas of Steroid Structures*, Vol. 1. New York: Plenum Press.

[bb4] Farrugia, L. J. (1997). *J. Appl. Cryst.* **30**, 565.

[bb5] Kant, R., Gupta, V. K., Kapoor, K., Kumar, M., Mallesha, L. & Sridhar, M. A. (2012). *Acta Cryst.* E**68**, o2590–o2591. 10.1107/S1600536812033375 PMC3415028 22905015

[bb6] Oxford Diffraction (2010). *CrysAlis PRO* Oxford Diffraction Ltd, Yarnton, England.

[bb7] Rodrigues, V. Z., Foro, S. & Gowda, B. T. (2011). *Acta Cryst.* E**67**, o2891. 10.1107/S1600536811040876 PMC3247308 22219926

[bb8] Sheldrick, G. M. (2008). *Acta Cryst.* A**64**, 112–122. 10.1107/S0108767307043930 18156677

[bb9] Spek, A. L. (2009). *Acta Cryst.* D**65**, 148–155. 10.1107/S090744490804362X PMC2631630 19171970

